# On-treatment HBV DNA dynamics predict virological breakthrough in entecavir-treated HBeAg-positive chronic hepatitis B

**DOI:** 10.1371/journal.pone.0174046

**Published:** 2017-03-28

**Authors:** Yi-Jie Huang, Chi-Sen Chang, Yen-Chun Peng, Hong-Zen Yeh, Sheng-Shun Yang

**Affiliations:** 1 Division of Gastroenterology, Department of Internal Medicine, Taichung Veterans General Hospital, Taichung, Taiwan; 2 School of Medicine, Chung Shan Medical University, Taichung, Taiwan; 3 School of Medicine, National Yang-Ming University, Taipei, Taiwan; Centers for Disease Control and Prevention, UNITED STATES

## Abstract

**Background & aims:**

Virological breakthrough (VBT) could be a manifestation of chronic hepatitis B (CHB) in patients treated with long-term nucleot(s)ide analogues. We aimed to determine the association of on-treatment serum hepatitis B virus (HBV) DNA with VBT in HBeAg-positive CHB patients receiving entecavir (ETV) treatment.

**Methods:**

A retrospective cohort study, including 162 consecutive patients (95 men and 67 women; mean age, 43.1±13.4 years) with HBeAg-positive CHB treated with ETV for at least 48 weeks between August 2008 and May 2015, was conducted. Univariate and multivariate cox regression analysis were used to identify associations with VBT and clinical factors, including HBV DNA and HBeAg serum status.

**Results:**

Among the 162 ETV-treated HBeAg-positive CHB patients, eighteen patients (11.1%) experienced VBT (VBT group), whereas the other 144 patients were without VBT (non-VBT group). The cumulative rate of HBV DNA < 100 IU/mL in the VBT group and the non-VBT group at week 48 were 44.44% and 70.14%, and at week 96 were 58.33% and 92.56%, respectively (p = 0.015). The cumulative rate of HBeAg seroclearance in the VBT group and non-VBT group at week 48 and week 96 were statistically significant (p = 0.014). Multivariate analysis disclosed that failure to achieve HBeAg seroclearance were the factors significantly associated with VBT.

**Conclusions:**

Our results demonstrated that on-treatment HBV DNA could probably predict VBT in ETV-treated HBeAg-positive chronic hepatitis B patients. Failure to achieve HBeAg seroclearance was associated with VBT in ETV-treated HBeAg-positive CHB patients. HBV DNA >100IU/mL at 48 weeks is potentially a predictor for VBT.

## Introduction

The standard treatment strategies for chronic hepatitis B (CHB) include lamivudine, adefovir, entecavir (ETV), telbivudine, tenofovir, and pegylated interferon. Recent studies demonstrated that antiviral therapy could ameliorate liver injury, progression of cirrhosis, and the development of HCC, and could also reduce hepatocellular carcinoma (HCC) recurrence among patients with HBV-related HCC.[1–3]

Virological breakthrough (VBT) could be the most important clinical hint for long-term nucleos(t)ide (NUC)-treated hepatitis B patients with drug resistance.[4, 5] The most common causes of VBT in NUC-treated CHB patients are genotypic resistance and medication adherence.[4, 6, 7] Once resistance confirmed, the treatment strategy is shifted to high potency antiviral agent without cross-resistance should be added as soon as possible.[4, 8] Thus, the standard of care involves on-treatment monitoring of data, including hepatic panel, hepatitis markers and serum HBV DNA, which were also recommended to be used as follow-up the treatment responses.[5, 9, 10]Now, HBV DNA is the standard and important tools for follow-up during NUC treatment. On-treatment HBV DNA monitoring is associated with the effect of treatment, including HBsAg and HBeAg seroclearance/seroconversion, drug resistance, and determination of cessation long-term NA treatment.[5, 9, 11]

ETV is a high-potency NUC capable of achieving viral suppression and resistance to ETV is rarely observed in long-term treatment-naïve patients. [12, 13]However, the association with dynamic change of on-treatment HBV DNA and VBT in ETV-treated CHB patients has not been clearly elucidated. Thus, the aim of this study was to determine the factors associated with VBT in ETV-treated CHB. We aimed to determine the association of on-treatment HBV DNA and the occurrence of VBT during ETV treatment in HBeAg-positive CHB patients.

## Materials and methods

### Patients

We conducted a retrospective cohort study including consecutive naive adult patients with CHB, who had received NUC treatment and whose treatment protocol was followed up regularly. Between August 2008 and May 2015, a total of 162 consecutive ETV-treated HBeAg-positive CHB patients (95 men and 67 women; mean age, 43.1±13.4 years) who were followed up for every 12–24 weeks were included in this study. The patients' demographics were regularly recorded as a hepatitis B treatment protocol, composing underlying diseases including liver cirrhosis, fatty liver, body weight, hepatitis B e antigen (HBeAg), HBV DNA, hepatic panel (albumin [Alb], alanine aminotransferase [ALT], total bilirubin (Bil-T), and alkaline phosphatase (ALP), platelet count, prothrombin time (PT), spleen size, renal function test including Creatinine Clearance Rate (CCr), and alpha fetoprotein (AFP). HBV DNA and HBeAg were routinely assessed every 3–6 months at Taichung Veterans General Hospital.

Patients were excluded if they had previous antiviral treatment for hepatitis B, HBeAg seroclearance within 24 weeks after ETV treatment, coinfection with hepatitis C virus, hepatitis D virus, or human immunodeficiency virus. The study was approved by the Institutional Review Board of our institution (VGHTC CE16037B).

### Definition of virological breakthrough

The occurrence of VBT, defined as any increase in serum HBV DNA by >1 log10 from nadir or redetection of serum HBV DNA at levels 10-fold the lower limit of detection of the viral load after HBV DNA was undetectable. Genotypic resistance (GR) was defined as detection of signature resistance mutation by direct sequencing.

### Laboratory methods

HBV DNA was determined by real-time PCR assay (Roche CobasTaqMan HBV Test). HBsAg and HBeAg were determined by electrochemiluminescence immunoassay (Roche Diagnostics, Mannheim, Germany).

### Tests for antiviral drug resistance mutations

HBV DNA was extracted from serum sample of patients by High Pure Viral Nucleic Acid Kit (Roche) and then determined by real-time PCR assay (QuantiTect SYBR Green PCR Kit)(QiaGene) for mutation detection. All samples were also tested for antiviral drug resistance mutations by a line probe assay, INNO-Lipa HBV DR v.3 (Innogenetics NV, Gent, Belgium) according to the manufacturer’s instructions.[14]

### Statistical analysis

Statistical tests were performed by SPSS (version 13.0; Chicago, IL, USA). Categorical variables were compared by Chi-squared test. Continuous variables were expressed as mean±SD, and compared by Mann–Whitney U test. The cumulative rates of HBeAg seroclearance and HBV DNA <100 IU/mL associated with VBT were calculated using the Kaplan–Meier method. The log rank test was used to compare the cumulative rate of HBeAg seroclearance and HBV DNA<100 IU/mL in different groups; Cox proportional hazard model was performed to analyze factors associated with VBT, and significant factors(P < 0.05) in the univariate analysis were subjected to multivariate analysis to determine independent predictive factors. Statistical significance was defined as a P value of less than 0.05.

## Results

### Patients' characteristics

**Fig 1:**
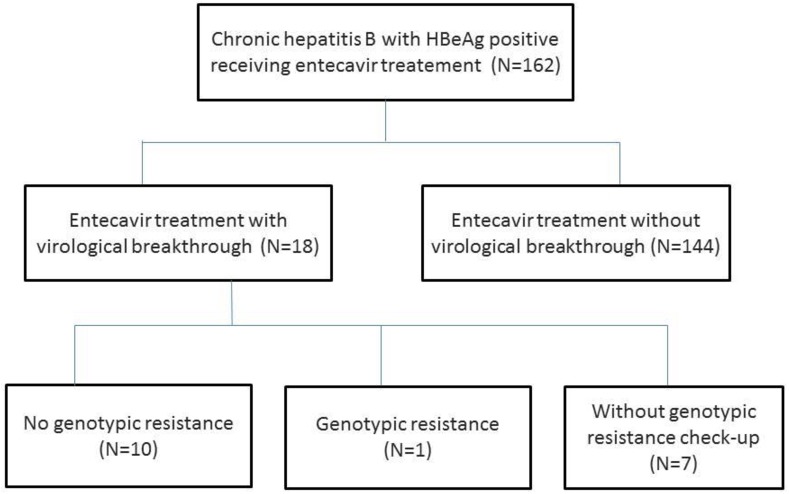
Outcome of patients with virological breakthrough.

The demographic data of ETV-treated HBeAg-positive CHB patients with VBT and without VBT are shown in Table 1. A total of 162 naïve HBeAg-positive CHB patients received ETV treatment. Eighteen (11.11%) ETV-treated patients experienced VBT and the other 144 (88.8%) patients did not experience VBT during follow-up. Only one (5.5%) of the VBT-experienced patients had confirmed GR with L180M and M204V mutation (S1 Table and S1 Fig). Among the VBT-experienced patients, 10 patients (55%) had no GR. Another 7 patents (38.8%) did not receive mutation check-up (Fig 1); 1 patient suspected of poor compliance according to medical record,3 patients experienced VBT with low serum HBV DNA (less than 130 IU/mL); 3 patients was loss to follow-up. There were no significant differences in gender, fatty liver, and liver cirrhosis between the two groups. There were significant differences in age between the VBT group and non-VBT group (p = 0.016). The baseline laboratory data including pre-treatment HBV DNA level, platelet count, spleen size, ALT, ALP, total bilirubin, PT, Alb, CCr, and AFP were not significantly different between the two groups.

**Table 1:** Baseline clinical characteristics of patients.

	Total (n = 162)	Virologic breakthrough		*P* value
		Yes (n = 18)	No (n = 144)	
Age	41.5 (34.0–52.0)	52 (38.5–59.5)	41 (33–49)	0.016*
Gender(Male)	95 (58.6%)	84 (58.3%)	11 (61.1%)	1.000
Follow-up time (weeks)	192 (140–250)	187.5 (104–233)	192 (144–254)	0.197
Fatty liver	66 (40.7%)	6 (33.3%)	60 (41.7%)	0.672
Liver cirrhosis ^f^	33 (20.5%)	4 (22.2%)	29 (20.3%)	0.766
Pre-treatment HBVDNA(log10 /mL)	7.53 (6.26–8.04)	7.33 (6.09–8.06)	7.53 (6.29–8.04)	0.817
Body weight (kg)	63 (55–77)	65 (51.2–84.5)	63 (55.0–76.7)	0.985
Spleen size (cm)	9.6 (8.4–11.4)	10.9 (8.6–12.5)	9.5 (8.4–11.2)	0.234
Platelet (*103/CUMM)	178 (127.0–228.5)	168 (118.5–245.5)	182 (127.5–228.0)	0.758
Total bilirubin (mg/dl)	0.8 (0.5–1.1)	0.7 (0.45–1.1)	0.8 (0.6–1.1)	0.551
ALT (U/L)	136 (69–367)	158 (74–370)	133 (68.-371)	0.852
ALP (U/L)	116 (89–156)	102 (90–140)	118 (87–160)	0.545
Albumin (g/dl)	3.9 (3.6–4.3)	3.8 (3.7–4.2)	4.0 (3.5–4.3)	0.698
Prothrobin time (s)	11.1 (10.6–11.6)	10.9 (10.2–11.5)	11.1 (10.6–11.7)	0.384
CCr (ml /min/1.73 m^2^)	92.56 (79–113.67)	104 (86–127)	91 (77–114)	0.107
AFP (ng/ml)	7.91 (4.33–16.46)	7.05 (5.79–11.23)	7.96 (4.185–16.735)	0.661

Chi-square test.

^f^ Fisher's Exact Test. Mann-Whitney U test.

*P<0.05

**P<0.01.

Continuous data were expressed median and IQR.

Categorical data were expressed number and percentage.

### Multivariate analysis of association with virological breakthrough

Table 2 shows the univariate and multivariate analysis of association with VBT. In the univariate analysis, failure to achieve HBV DNA <100 IU/mL at week 48 and nadir response, HBeAg seroclearance and age were associated with VBT. The multivariate analysis showed HBeAg seroclearance was significantly associated with VBT (HR = 0.25, CI = 0.07–0.94, p = 0.04)(Table 2)

**Table 2:** Univariate and multivariate analyses of factors associated with virological breakthrough.

	Univariate analysis			Multivariate analysis		
	HR	95% CI	*P* value	HR	95% CI	*P* value
Pre-treatment HBVDNA level (log IU/ml)	1.05	(0.70–1.57)	0.818	0.86	(0.52–1.41)	0.545
Post-treatment HBV DNA<100IU/mL	0.04	(0.01–0.12)	<0.001**			
Post-treatment HBV DNA<100IU/mL at week 48	0.32	(0.12–0.80)	0.015*	0.32	(0.10–1.00)	0.050
HbeAgseroclearance	0.17	(0.05–0.60)	0.006*	0.25	(0.07–0.94)	0.040*

Cox regression.

Adjusted for age, gender, body weight, pre-treatment HBVDNA level (log IU/ml), post-treatment HBV DNA<100IU/mL at week 48,HbeAgseroclearance.

*P<0.05

**P<0.01.

### Characteristics and clinical outcome of patients experienced virological breakthrough

Information on baseline characteristics and clinical outcome of 18 ETV-treated HBeAg-positive CHB patients with VBT is provided in Table 3. Of the 18 patients who experienced VBT, 5 patients continued treatment with ETV, 8 patients received add-on ADF to ETV and 5 patients received TDF as rescue therapy. Regarding to the outcome of these patients, 10 patients achieved VR, 4 patient died, 3 patients lost to on-treatment follow-up and 1 patient did not achieve to VR.

**Table 3:** Baseline characteristics and clinical outcome of patients experienced virological breakthrough.

Patient No	Age (yr)	Sex	Mutation	Rescue therapy	Clinical outcome
1	39	F	─	Maintenance of ETV	Death
2	67	M	─	ETV and ADF	Achieved VR
3	52	F	ND	ETV and ADF	Achieved VR
4	33	M	ND	Maintenance of ETV	Loss of follow-up
5	54	M	L180M, M204V	ETV and ADF	Death
6	54	M	ND	TDF	Achieved VR
7	49	F	─	Maintenance of ETV	Loss of follow-up
8	71	F	ND	TDF	Achieved VR
9	61	M	ND	ETV and ADF	Death
10	59	F	ND	TDF	Achieved VR
11	59	M	─	ETV and ADF	Achieved VR
12	41	M	ND	ETV and ADF	Achieved VR
13	31	M	─	Maintenance of ETV	Loss of follow-up
14	71	F	ND	TDF	Achieved VR
15	41	M	─	Maintenance of ETV	Failure to VR
16	53	M	ND	TDF	Death
17	36	F	ND	ETV and ADF	Achieved VR
18	37	M	─	ETV and ADF	Achieved VR

─,No data available

ND, mutation was not dectected

VR, viral suppression; HBV DNA < 100IU/mL

ETV = Entecavir; ADF = Adefovir; TDF = Tenofovir

### On-treatment HBV DNA association with virological breakthrough

**Fig 2:**
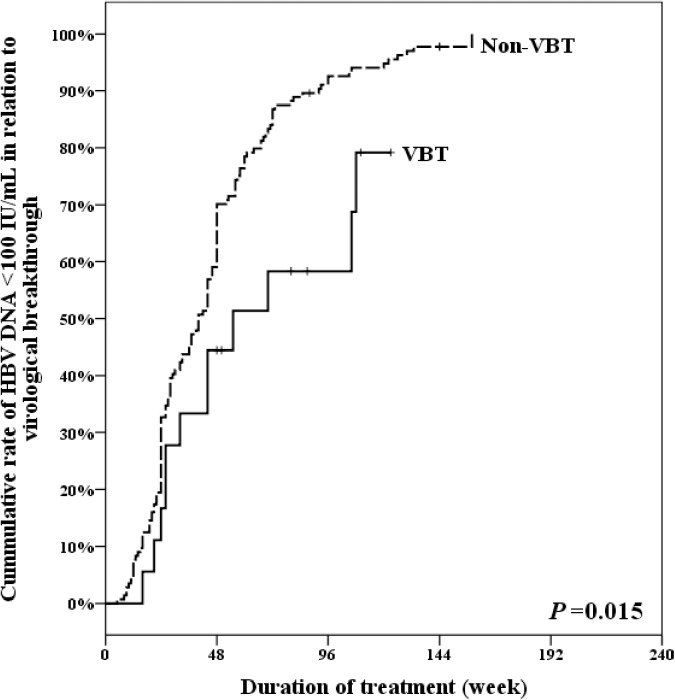
Cumulative rate of HBV DNA <100IU/mL in relation to virological breakthrough. VBT: virological breakthrough.

Our results demonstrated that the cumulative rates of HBV DNA < 100 IU/mL in the VBT group and non-VBT group at week 48 were44.44% and 70.14%, and at week 96 were 58.33% and 92.56%, respectively (p = 0.015) (Fig 2).

### On-treatment HBeAg seroclearance association with virological breakthrough

**Fig 3:**
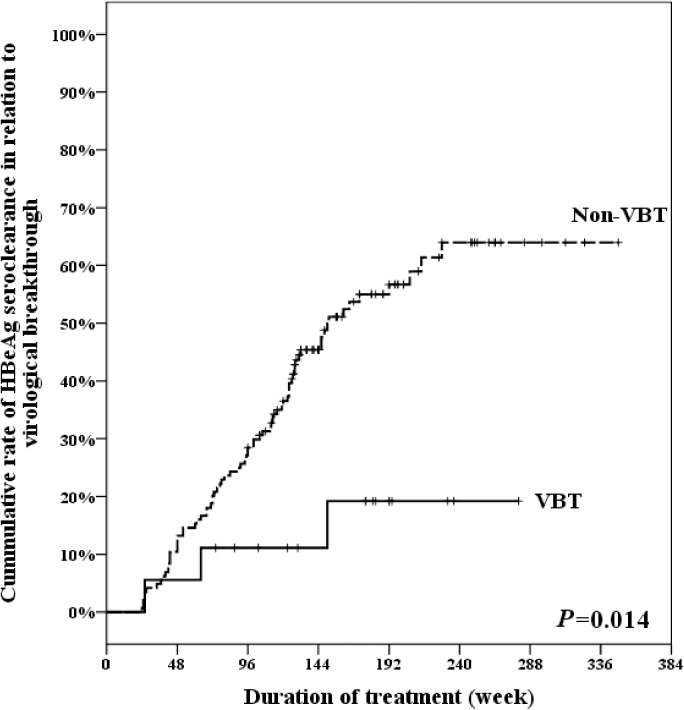
Cumulative rate of HBeAg seroclearance in relation to virological breakthrough. VBT: virological breakthrough.

Our results demonstrated that cumulative rate of HBeAg seroclearance in the VBT group and non-VBT group at weeks 48,96,144,192, and 240 were 5.56% and 13.19%,11.11% and 28.47%,11.11% and 45.37%,19.19% and 56.68%,19.19% and 63.95%, respectively (p = 0.014) (Fig 3).

## Discussion

Our results demonstrated 18 (11.1%) of 162 patients with ETV-treated HBeAg-positive CHB experienced VBT. HBeAg seroclearance was associated with a lower possibility of VBT, as demonstrated by our results. Among the 18 patients who had VBT and were continuously followed up, failure to achieve HBeAg seroclearance was independent predictors for VBT. Failure to achieve HBV DNA <100IU/mL within 48 weeks is potentially a predictor for VBT in ETV-treated HBeAg-positive CHB patients. This finding implies that we could use on-treatment HBV DNA and HBeAg seroclearance to assess treatment and predict the likelihood of VBT.

The decline of on-treatment HBV DNA is important in the assessment of the treatment response. Serum HBV DNA level >2000 IU/mL after 6 months of lamivudine therapy is associated with the occurrence of VBT in CHB patients and a decrease in follow-up HBV DNA levels (> 3 log) was significantly associated with HBsAg seroclearance.[11, 15]The 2-Year GLOBE trial results revealed baseline HBV DNA and on-treatment HBV DNA at week 24 were risk factors associated with VBT in CHB patients receiving lamivudine and telbivudine. [16] Poor response to viral suppression could be correlated with occurrence of drug-resistance. Non-detectable serum HBV DNA at treatment week 24 was the strongest predictor for HBeAg seroconversion, virological breakthrough, and virus resistance.[15, 17]Patients with on-treatment HBV DNA levels of more than 1000 copies/mL after 6 months of lamivudine therapy there was a 63.2% chance of subsequent occurrence of YMDD variants.[18] Thus, we could use on-treatment HBV DNA level to predict the treatment effect as well as VBT. To our best knowledge, there are scant data on ETV-treated naïve HBeAg-positive patients, and the present study is the first to describe the association of HBV DNA and HBeAg dynamics with VBT in ETV-treated HBeAg-positive patients.

ETV is currently the first-line therapy due to its potent viral suppression capability and the rarity of ETV-resistant mutations.[12, 13, 19] ETV has also been used as a rescue treatment option for patients with a high genetic barrier.[4, 20] However, the efficacy of ETV is affected by on-treatment virological response and failure to achieve virological response at week 48 is a strong predictor of ETV-resistant mutation, especially in patients with lamivudine-resistant mutants.[21] Cumulative rate of VBT at week 96 and 144 was higher in CHB patients with partial virological response compared to patients with complete virological response under ETV treatment.[22] The cumulative probability of genotypic resistance and virological breakthrough due to resistance were 51% and 43% at year 5, respectively, in ETV-treated patients with mutation of rtM204I/V and rtl180 M.[12]

Patients with CHB usually require long-term antiviral agents due to the low rate HBsAg seroclearance, HBeAg seroconversion, and HBV DNA clearance. [9] Thus, it is important to monitor VBT during follow-up. The main causes of VBT included medical adherence and genotype resistance. The first step is to differentiate between VBT due to poor drug compliance and VBT due to drug resistance. About 60% of VBTs are related to drug resistance. Confirmation of medication adherence and antiviral mutation in CHB is important to avoid unnecessary change of antiviral therapy.[23] Thus, VBT could be a valuable parameter in the clinical assessment of antiviral drug resistance as it typically appears within 3 months of the emergence of resistance.[18]Furthermore, it would be particularly useful to closely monitor VBT in patients with persistent higher HBVDNA level, poor drug compliance, and inadequate medical adherence. HBV DNA is not only used to monitor treatment response but also for surveillance of the possibility of antiviral resistance because the use of routine genotypic testing is not practical due to the high cost. Once resistance to ETV is confirmed, therapeutic options include adding adefovir or switching to drugs with a higher potency and higher genetic barriers to resistance, such as tenofovir.[4]

There are some limitations in the present study. First, it is difficult to differentiate HBeAg seroclearance within 6 months of entecavir due to efficacy of antiviral therapy and due to spontaneous HBeAg seroclearance. So patients with HBeAg seroclearance within 6 months of entecavir should be excluded. Therefore, the number of cases was small and all patients were HBeAg-positive. Further studies may be needed to investigate the association between VBT and other clinical outcomes in CHB patients who are HBeAg-negative. Second, as a retrospective study, presence of antiviral drug resistance mutations was only checked in 11 of 18 (61%) patients. Third, because this was a retrospective study, adherence of drugs could not be definitely confirmed by reviewing the medical record. Fourth, there was lack of quantitative HBsAg level in this study.

In conclusion, our results demonstrated that on-treatment HBV DNA dynamics could predict VBT in ETV-treated HBeAg-positive chronic hepatitis B patients. The clearance of serum HBeAg was associated with VBT in ETV-treated HBeAg-positive CHB patients. On-treatment HBV DNA could predict VBT in ETV-treated HBeAg-positive CHB patients and further studies are needed to elucidate this potential association. Naïve HBeAg-positive CHB patients receiving ETV with VBT had a low HBeAg seroclearance rate and failed to achieve a low serum HBV DNA level during follow-up.

## Supporting information

S1 Fig Alignment of amino acid sequence of hepatitis B virus DNA in the patient with mutation. (TIF)

S1 Table The result of mutations detectable. (DOCX)

S1 File Database. (XLSX)

## References

[1] Jang JW, Choi JY, Kim YS, Woo HY, Choi SK, Lee CH, et al Long-term effect of antiviral therapy on disease course after decompensation in patients with hepatitis B virus-related cirrhosis. Hepatology (Baltimore, Md). 2015;61(6):1809–20. Epub 2015/01/30. 10.1002/hep.27723 25627342

[2] Papatheodoridis GV, Chan HL, Hansen BE, Janssen HL, Lampertico P. Risk of hepatocellular carcinoma in chronic hepatitis B: assessment and modification with current antiviral therapy. Journal of hepatology. 2015;62(4):956–67. Epub 2015/01/18. 10.1016/j.jhep.2015.01.002 25595883

[3] Wu CY, Chen YJ, Ho HJ, Hsu YC, Kuo KN, Wu MS, et al Association between nucleoside analogues and risk of hepatitis B virus-related hepatocellular carcinoma recurrence following liver resection. Jama. 2012;308(18):1906–14. Epub 2012/11/20. 23162861 10.1001/2012.jama.11975

[4] Ghany MG, Doo EC. Antiviral resistance and hepatitis B therapy. Hepatology (Baltimore, Md). 2009;49(5 Suppl):S174–84. Epub 2009/04/29. 10.1002/hep.22900 PubMed Central PMCID: PMCPmc2707848. PMC2707848 19399794

[5] Lok AS, McMahon BJ. Chronic hepatitis B: update 2009. Hepatology (Baltimore, Md). 2009;50(3):661–2. Epub 2009/08/29. 10.1002/hep.23190 . 19714720

[6] Chotiyaputta W, Peterson C, Ditah FA, Goodwin D, Lok AS. Persistence and adherence to nucleos(t)ide analogue treatment for chronic hepatitis B. Journal of hepatology. 2011;54(1):12–8. Epub 2010/10/05. 10.1016/j.jhep.2010.06.016 20888661

[7] Liaw YF, Leung N, Kao JH, Piratvisuth T, Gane E, Han KH, et al Asian-Pacific consensus statement on the management of chronic hepatitis B: a 2008 update. Hepatology international. 2008;2(3):263–83. Epub 2009/08/12. PubMed Central PMCID: PMCPmc2716890. 10.1007/s12072-008-9080-3 19669255 PMC2716890

[8] Bang KB, Kim HJ. Management of antiviral drug resistance in chronic hepatitis B. World Journal of Gastroenterology: WJG. 2014;20(33):11641–9. 10.3748/wjg.v20.i33.11641 25206270 PMC4155356

[9] European Association for the Study of the L. EASL Clinical Practice Guidelines: Management of chronic hepatitis B virus infection. Journal of hepatology. 57(1):167–85. 10.1016/j.jhep.2012.02.010 22436845

[10] Keeffe EB, Zeuzem S, Koff RS, Dieterich DT, Esteban-Mur R, Gane EJ, et al Report of an international workshop: Roadmap for management of patients receiving oral therapy for chronic hepatitis B. Clinical gastroenterology and hepatology: the official clinical practice journal of the American Gastroenterological Association. 2007;5(8):890–7. Epub 2007/07/17. 10.1016/j.cgh.2007.05.004 . 17632041

[11] Liu J, Yang HI, Lee MH, Lu SN, Jen CL, Wang LY, et al Incidence and determinants of spontaneous hepatitis B surface antigen seroclearance: a community-based follow-up study. Gastroenterology. 2010;139(2):474–82. Epub 2010/05/04. 10.1053/j.gastro.2010.04.048 20434450

[12] Tenney DJ, Rose RE, Baldick CJ, Pokornowski KA, Eggers BJ, Fang J, et al Long-term monitoring shows hepatitis B virus resistance to entecavir in nucleoside-naive patients is rare through 5 years of therapy. Hepatology (Baltimore, Md). 2009;49(5):1503–14. Epub 2009/03/13. 10.1002/hep.22841 19280622

[13] Keeffe EB, Dieterich DT, Pawlotsky JM, Benhamou Y. Chronic hepatitis B: preventing, detecting, and managing viral resistance. Clinical gastroenterology and hepatology: the official clinical practice journal of the American Gastroenterological Association. 2008;6(3):268–74. Epub 2008/03/11. 10.1016/j.cgh.2007.12.043 . 18328434

[14] Degertekin B, Hussain M, Tan J, Oberhelman K, Lok AS. Sensitivity and accuracy of an updated line probe assay (HBV DR v.3) in detecting mutations associated with hepatitis B antiviral resistance. Journal of hepatology. 2009;50(1):42–8. Epub 2008/11/21. 10.1016/j.jhep.2008.08.020 19019484

[15] Su CW, Wu CY, Hung HH, Wu CH, Sheen IJ, Wu JC. Differential roles of serum hepatitis B virus DNA and hepatitis B surface antigen level in predicting virological breakthrough in patients receiving lamivudine therapy. Journal of gastroenterology and hepatology. 2013;28(12):1849–58. Epub 2013/06/05. 10.1111/jgh.12283 23730852

[16] Liaw YF, Gane E, Leung N, Zeuzem S, Wang Y, Lai CL, et al 2-Year GLOBE trial results: telbivudine Is superior to lamivudine in patients with chronic hepatitis B. Gastroenterology. 2009;136(2):486–95. Epub 2008/11/26. 10.1053/j.gastro.2008.10.026 19027013

[17] Zeuzem S, Gane E, Liaw YF, Lim SG, DiBisceglie A, Buti M, et al Baseline characteristics and early on-treatment response predict the outcomes of 2 years of telbivudine treatment of chronic hepatitis B. Journal of hepatology. 2009;51(1):11–20. Epub 2009/04/07. 10.1016/j.jhep.2008.12.019 19345439

[18] Yuen MF, Sablon E, Hui CK, Yuan HJ, Decraemer H, Lai CL. Factors associated with hepatitis B virus DNA breakthrough in patients receiving prolonged lamivudine therapy. Hepatology (Baltimore, Md). 2001;34(4 Pt 1):785–91. Epub 2001/10/05. 10.1053/jhep.2001.27563 . 11584376

[19] Pol S, Lampertico P. First-line treatment of chronic hepatitis B with entecavir or tenofovir in ‘real-life’ settings: from clinical trials to clinical practice. Journal of Viral Hepatitis. 2012;19(6):377–86. 10.1111/j.1365-2893.2012.01602.x 22571899 PMC3489060

[20] Petersen J, Ratziu V, Buti M, Janssen HL, Brown A, Lampertico P, et al Entecavir plus tenofovir combination as rescue therapy in pre-treated chronic hepatitis B patients: an international multicenter cohort study. Journal of hepatology. 2012;56(3):520–6. Epub 2011/11/01. 10.1016/j.jhep.2011.09.018 22037226

[21] Chen CH, Hu TH, Hung CH, Wang JH, Lu SN, Lee CM. Antiviral effect of entecavir in nucleos(t)ide analogue-naive and nucleos(t)ide analogue-experienced chronic hepatitis B patients without virological response at week 24 or 48 of therapy. J Viral Hepat. 2014;21(8):e55–64. Epub 2014/04/29. 10.1111/jvh.12239 24766327

[22] Kwon DH, Kim IH, Choung BS, Ahn DS, Yoo SH, Park SB, et al Continuous Long-Term Entecavir Therapy in Naïve Chronic Hepatitis B Patients Showing Partial Virologic Response. Gut and Liver. 2013;7(6):712–8. 10.5009/gnl.2013.7.6.712 24312713 PMC3848542

[23] Hongthanakorn C, Chotiyaputta W, Oberhelman K, Fontana RJ, Marrero JA, Licari T, et al Virological breakthrough and resistance in patients with chronic hepatitis B receiving nucleos(t)ide analogues in clinical practice. Hepatology (Baltimore, Md). 2011;53(6):1854–63. Epub 2011/05/28. 10.1002/hep.24318 . 21618260

