# Supplementary figures and images for: On-treatment HBV DNA dynamics predict virological breakthrough in entecavir-treated HBeAg-positive chronic hepatitis B
Source: PLoS One. 2017 Mar 28;12(3):e0174046. doi: 10.1371/journal.pone.0174046 (PMC5369759; doi:10.1371/journal.pone.0174046)

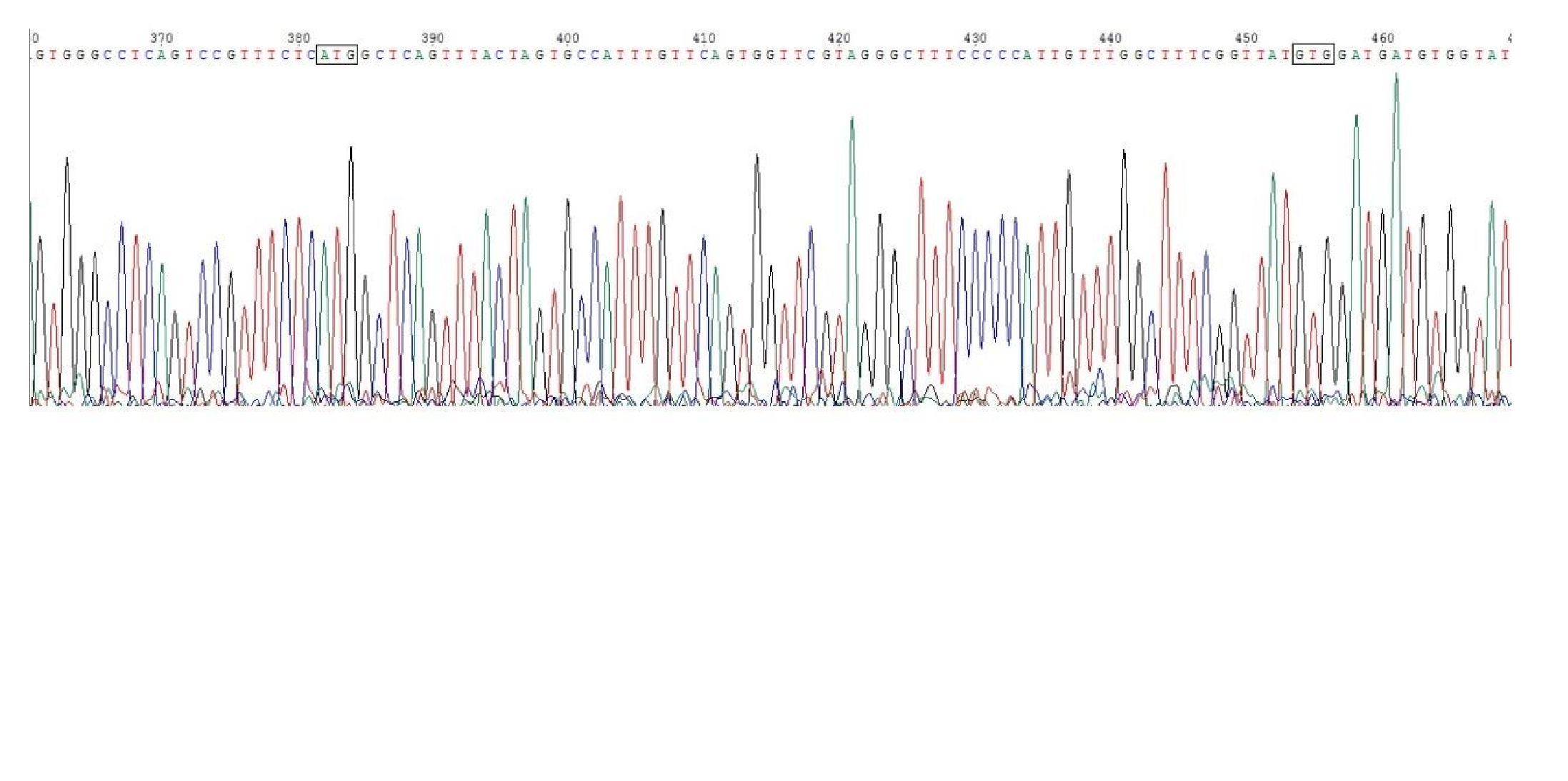

Supplement: S1 Fig — (TIF) [file pone.0174046.s001.tif]
