# Supplementary material for: On-treatment HBV DNA dynamics predict virological breakthrough in entecavir-treated HBeAg-positive chronic hepatitis B
Source: PLoS One. 2017 Mar 28;12(3):e0174046. doi: 10.1371/journal.pone.0174046 (PMC5369759; doi:10.1371/journal.pone.0174046)
Supplement: S1 Table — (DOCX) [file pone.0174046.s002.docx]

| Supplementary Table 1 : The result of mutations detectable | | | | | | |  |  |
| --- | --- | --- | --- | --- | --- | --- | --- | --- |
| Mutation analysis | | amino acid 80: Leucine→ Leucine | | | | |  |  |
|  |  | amino acid 173: Valine → Valine | | | | |  |  |
|  |  | **amino acid 180: Leucine → Methionine** | | | | |  |  |
|  |  | amino acid 181: Alanine →Alanine | | | | |  |  |
|  |  | **amino acid 204: Methionine →Valine** | | | | |  |  |
|  |  |  |  |  |  |  |  |  |
|  |  |  |  |  |  |  |  |  |
|  |  |  |  |  |  |  |  |  |
|  |  |  |  |  |  |  |  |  |
